# The validation of a new measure quantifying the social quality of life of ethnically diverse older women: two cross-sectional studies

**DOI:** 10.1186/1471-2318-11-60

**Published:** 2011-10-08

**Authors:** Luciana Laganà, Maria L Bratly, Ioakim Boutakidis

**Affiliations:** 1 Department of Psychology, California State University Northridge, 18111 Nordhoff Street, Northridge, California, USA; 2 Department of Child and Adolescent Studies, California State University Fullerton, 800 State College Boulevard, Fullerton, California, USA

## Abstract

**Background:**

To our knowledge, the available psychometric literature does not include an instrument for the quantification of social quality of life among older women from diverse ethnic backgrounds. To address the need for a tool of this kind, we conducted two studies to assess the initial reliability and validity of a new instrument. The latter was created specifically to quantify the contribution of a) social networks and resources (e.g., family, friends, and community) as well as b) one's perceived power and respect within family and community to subjective well-being in non-clinical, ethnically diverse populations of older women.

**Methods:**

In Study 1, we recruited a cross-sectional sample of primarily non-European-American older women (*N *= 220) at a variety of community locations. Participants were administered the following: a short screener for dementia; a demographic list; an initial pool of 50 items from which the final items of the new Older Women's Social Quality of Life Inventory (OWSQLI) were to be chosen (based on a statistical criterion to apply to the factor analysis findings); the Single Item Measure of Social Support (SIMSS); and the Medical Outcome Study 36-item Short-Form Health Survey (MOS SF-36). Study 2 was conducted on a second independent sample of ethnically diverse older women. The same recruitment strategies, procedures, and instruments as those of Study 1 were utilized in Study 2, whose sample was comprised of 241 older women with mostly non-European-American ethnic status.

**Results:**

In Study 1, exploratory factor analysis of the OWSQLI obtained robust findings: the total variance explained by one single factor with the final selection of 22 items was over 44%. The OWSQLI demonstrated strong internal consistency (*α *= .92, *p *< .001), adequate criterion validity with the SIMSS (*r *= .33; *p *< .01), and (as expected) moderate concurrent validity with the MOS SF-36 for both physical (*r *= .21; *p *< .01) and mental (*r *= .26; *p *< .01) quality of life. In order to confirm the validity of the 22-item OWSQLI scale that emerged from Study 1 analyses, we replicated those analyses in Study 2, although using confirmatory factor analysis. The total variance accounted for by one factor was about 42%, again quite high and indicative of a strong single-factor solution. Study 2 data analyses yielded the same strong reliability findings (i.e., *α *= .92, *p *< .001). The 22-item OWSQLI was correlated with the SIMSS (*r *= .27, *p *< .001) in the expected direction. Finally, correlations with the MOS SF- 36 demonstrated moderate concurrent validity for physical (*r *= .14; *p *< .01) and mental (*r *= .18; *p *< .01) quality of life, as expected.

**Conclusions:**

The findings of these two studies highlight the potential for our new tool to provide a valid measure of older women's social quality of life, yet they require duplication in longitudinal research. Interested clinicians should consider using the OWSQLI in their assessment battery to identify older women's areas of lower versus higher social quality of life, and should establish the maximization of patients' social quality of life as an important therapeutic goal, as this variable is significantly related to both physical and mental health.

## Background

The growing diversity of aging populations and corresponding expectations of successful aging have motivated multidisciplinary investigations of quality of life (abbreviated as QofL herein). Several researchers have highlighted the need to develop assessment tools to properly quantify psychosocial variance in older age [1-4]. Research on social facets of QofL in later life may contribute to an increased understanding of protective social resources that could enhance the biopsychosocial health of older adults. In turn, this process could assist in addressing the aging population's health care impact on public resources, leading to a more cost-effective use of the health care system [5-7]. The social QofL of older women in particular should be a topic of focused investigation because this population, compared to older men, typically seeks medical help more often, experiences less than adequate assistance with basic life activities, and reports fewer financial resources [8-10]. Considering the aforementioned disadvantages, maximizing older women's social QofL (assessed using a methodologically appropriate measure) is critical.

The focus of the present research is the initial validation of a new social QofL tool, which is intended for use with older women from diverse ethnic backgrounds. Ethnic minorities in general are understudied in QofL research for a variety of reasons. Several researchers have identified factors such as low socioeconomic status [11,12] (a characteristic of many ethnically diverse older women [10]) and limited English-language proficiency [13] as barriers to the inclusion of ethnic minority groups in QofL studies. Also, scarcity of culturally-competent (preferably same-culture) researchers is often problematic [14]. In addition, older women's limited financial means can present difficulties regarding transportation to research sites [15]. We have minimized these challenging issues in the current research, as described in the Methods section. There is ample evidence on the contribution of social variables (such as the need to feel loved [16,17] as well as respected and valued by others [18,19]) to the well-being of older women (and men). Many such issues hold unique relevance among older individuals from ethnic minority backgrounds due to the emphasis placed upon family, friends, and the community at large, as they typically favor a collectivistic orientation [20].

In research on older women, a satisfying social life has been related to better physical health. For instance, in a study on 471 women over the age of 60 living with cardiac disease, respondents in recovery from cardiac events who had satisfactory social resources displayed fewer depressive symptoms and better coping skills [21]. Moreover, those who reported greater social support exhibited enhanced emotional well-being, lower symptom impact, and greater perceived health. Social networks could produce satisfying and trusting relationships with family and close friends and, importantly, enhance older women's ability to compensate for physical functioning deficits by reducing levels of distress [23]. Indeed, mental health is significantly related to satisfaction with one's social life and resources. Numerous studies have confirmed the positive role of social support, integration, and engagement in providing psychosocial benefits for improved mental well-being and QofL in later life [5,24-26]. Additionally, Jang, Haley, Small, and Mortimer [22], in a study of 406 women (60 years old and older) diagnosed with cardiac disease, discovered that social resources enhanced psychological resilience. Social integration may also protect older adults from cognitive decline by positively influencing social competence and mood [27]. Furthermore, emotional and social expectations attached to social interactions afford a perception of continued value, meaningful connections to others, and emotional support for reassurance and affection, which contribute to morale and satisfaction in older age [28,29]. In addition, social resources are associated with enhanced feelings of self-esteem, which may strengthen older adults' coping skills and lessen the impact of negative life events, losses, and functional decline [22,30].

### The Need for a Measure of Older Women's Social QofL

Due to the lack of an appropriate measure to quantify social QofL in older women, to date, researchers interested in assessing psychosocial aspects of well-being in this population have relied mostly on health-related QofL measures that were typically not designed to capture the increasing diversity of older populations. Furthermore, by definition, all health-related QofL instruments confound physical health status with social well-being, if they assess the latter at all. Given the advanced age of our target population and the corresponding high potential for health problems and functional limitations that could extend to all areas of older women's lives, instruments need to be developed for the quantification of social QofL independently from physical/medical symptomatology. This task should be accomplished with a measure that is designed to be sensitive to culturally-relevant social variables, given the ethnic diversity of older women populations. However, few researchers have targeted the above-mentioned QofL issues within non-clinical older populations from diverse ethnic backgrounds [31]. In fact, a web-based search for this kind of tool resulted in few qualified measures. What follows is a brief review of some of the popular QofL scales, organized by their limitations in assessing social QofL in the target population of the present research.

### Researchers Must Be Able to Discriminate Between Physical and Social QofL

Most of the commonly used measures confound socio-emotional QofL with physical/medical health. For example, the utilization of the *Medical Outcome Study 36-Item Short-Form Health Survey (MOS SF-36) *[32] allows the quantification of both physical and mental health components of QofL. While researchers who implemented this measure have reported good sensitivity as well as cross-cultural generalizability [33-35], the MOS SF-36 was constructed mainly to quantify limitations in social (and other) activities caused by health problems, thus confounding general health status and functional loss with QofL. Similarly, the *Quality of Life Index - Generic Version (QLI) *[36] and the *World Health Organization's QofL assessment (WHOQofL-100) *[37] confound social aspects of QofL (such as social and family functioning) with health status.

### Assessment Tools Must Allow Generalization of Findings to Non-Clinical Populations

Measures designed specifically for use with clinical populations (including individuals with acute mental disorders or medical diagnoses) may not be sensitive to minor changes in status within non-clinical populations. To offer an example, one of the earliest QofL assessments, the *Spitzer QL-Index *[38] has been shown to effectively assess general health, social support, and psychological outlook. However, this assessment tool was validated on cancer patients in palliative care. A scale designed to be relevant to individuals with terminal illness may not be sufficiently discriminatory or applicable to healthy--or relatively healthy--populations. Similarly, the 46-item *Schedule for the Evaluation of Individual Quality of Life (SEIQoL) *[39] and the *SEIQoL-DW15*-item version [40] were originally created to assess QofL in palliative care settings. Although empirical evidence seems to indicate that both measures are psychometrically sound, the appropriateness of their application for routine use in non-palliative care settings or with individuals without serious illness is dubious [41]. Similar criticism can be applied to the *Nottingham Health Profile (NHP) *[42] and the *Quality of Well-Being Scale (QWS) *[43,44], which primarily assess the impact of disease and illness, making their applicability to fairly healthy populations questionable.

### Measures Need to Be Validated on Ethnically Diverse Older Adult Populations

Few QofL measures have been explicitly normalized or psychometrically validated on ethnically diverse populations, which limits their external validity. Indeed, researchers have challenged assumptions of the universality of health-related assessment tools such as the MOS SF-36 [45]. In particular, Staniszewska, Ahmed, and Jenkinson discovered that cultural influences in White and Indian cardiac patients' descriptions of their physical health were similar, yet greater avoidance of terms such as "mental" and "emotional" surfaced among Indian participants. Additionally, different religious perceptions affected the interpretation of disease by these two groups. Deyo conducted a study [46] comparing the original English language version of the *Sickness Impact Profile (SIP) *[47] (a well-established self-report measure of physical QofL/functional health status) to a Spanish language version within a sample of non-Hispanic and Mexican-American participants with low back pain. The SIP produced results with English-speaking individuals that seemed highly valid, while findings for Mexican-American participants who used the English language SIP measure were poor. Mexican-American patients using the Spanish version of the SIP obtained the poorest results. This suggests that simply producing an adequate translation of the SIP does not address culturally unique responses to symptoms, such as the reluctance of some cultures to answer personal questions and the general effects that acculturation may produce in the comfort level experienced by individuals responding to scale items [45].

Many popular QofL measures appear to disregard older adults, a segment of the population for whom reliable assessment of the social components of QofL may be especially critical (for reasons briefly highlighted earlier). Unfortunately, current QofL measures are not well-designed to serve this population. For instance, Bowling stated that the MOS SF-36 does not cover sleep, economic welfare, sexual functioning, education, independence, or religion; these are all domains that influence QofL perceptions in later life [2]. Moreover, the QLI referenced earlier [36] is reportedly less sensitive to QofL change in older adults with chronic illness than the MOS SF-36 [34]. We identified a few instruments that cover social QofL issues and were used on older populations. However, a closer examination of such measures revealed many of the same limitations already discussed. For example, the *Quality of Life Scale for the Elderly (QLSE) *[48], which was ostensibly designed to quantify the social QofL of healthy older adults from non-clinical populations, was presented in a doctoral dissertation, but, to our knowledge, has not been used in peer-reviewed empirical research. More importantly, an examination of the content of the QLSE items reveals that this tool confounds social QofL with physical QofL. Another measure, the *Quality of Life Enjoyment and Satisfaction Scale (Q-LES-Q) *[49], covers social aspects of QofL. However, although it has been administered to older populations, it was not designed for older adults or older women in particular. Furthermore, all of the studies that we were able to identify on this scale covered clinical populations that presented some form of mental illness or disability [50-52].

### The Social QofL Measure Proposed Herein

The literature summarized above regarding available tools for the assessment of social QofL demonstrates a gap in the psychometric research on this topic concerning non-clinical populations of older women. An instrument constructed specifically for use with community-dwelling, ethnically diverse older women is critical to capturing the impact of familial and community support systems on these women's QofL, as well as their perception of power, respect, understanding, and honor within their family and community--all of which are concepts that are critical to social well-being in older age, as mentioned earlier. Before describing our two studies on this measure, it is appropriate to briefly illustrate their conceptual foundations. In a classic theorization (adopted as the conceptual framework of the present research), Engel [53,54] argued that the boundaries between health and disease are intertwined with social, medical, and psychological factors. Thus, one's perception of social well-being represents an essential component of health and should not be neglected in research; based on this theoretical approach, we compiled original items that could best assess social QofL in older women from diverse ethnic backgrounds. We focused on including in this measure items covering topics that emerged from the review of the existing literature on older women's social resources, particularly aspects of life related to their social ties (with family, extended family, friends, peers, neighbors, church members, and the community at large) that have been associated with QofL, yet not assessed via a single measure.

It should be noted that the new instrument proposed herein does not assess health-related QofL (to avoid confounding social QofL with health status), unlike most of the aforementioned tools such as the MOS SF-12/36, the QLI, the SIP, and the NHP, among others. Our social QofL measure quantifies instead the perceptions and reactions of older women to several facets of their environment (e.g., social and emotional). A similar conceptual framework provided the foundation for the *Life Satisfaction in the Elderly Scale *[55,56]. However, the latter scale is not gender-specific and confounds health status with QofL. To our knowledge, our new measure is the first tool created specifically to assess community-dwelling older women's social QofL beyond health status. We hypothesized that, in both studies, the new instrument would show strong factor analysis, reliability, and initial validity findings. Concerning the latter, we expected that scores on our measure would be moderately related to the size of respondents' support network. Moreover, we anticipated a significant relationship between social QofL and both kinds of QofL that have been most commonly targeted in prior studies, i.e., physical and mental. However, only a modest association with these two facets of QofL was hypothesized, given the specific social focus of the new tool.

## Methods

### Sample Characteristics

The ethnically diverse sample of Study 1 consisted of 220 older women who resided in several urban and suburban communities in Los Angeles County; each respondent self-identified as a member of one of seven ethnic/pan-ethnic groups. The demographic characteristics of the sample are displayed in Table 1. Participants ranged in age from 60 to 97 years, with a reported median age of 71 and an interquartile range of 66.25 to 78.00 years of age. A little over one-third of the sample was European-American; more than half indicated being born outside of the United States (U.S.). About 51% of the participants had a high school education or less. Most of the respondents were retired (and, therefore, unemployed) and mainly married or widowed. For Study 2, we recruited 241 older women who resided in the same urban and suburban communities of Los Angeles County as Study 1 participants. These women reported a median age of 68 years, with a total range of 60 to 90 years and an interquartile range of 64.00 to 75.00 years of age. In this second sample, women self-identified as belonging to one of seven ethnic/pan-ethnic groups. Table 2 illustrates the demographic characteristics of this sample. Approximately 40% of the participants identified as European-American and indicated an educational level equal to or lower than high school completion. Nearly half reported being born outside of the U.S. Inclusion criteria for both studies were as follows: 60 years of age or older (based on the age of many of the aforementioned studies' samples), English fluency (to minimize confounding the results with acculturation levels), ability to provide informed consent, and independent living status. Excluding individuals in assisted living facilities decreased the possibility of recruiting participants with significant cognitive impairment (who are often found in such institutions).

**Table 1:** Demographics Characteristics of Study 1 Sample (*N *= 220)

*Demographic variables*	*Median* *%*
Age	71
Ethnicity	
European-American	37.3
Latina/Hispanic	28.2
Middle Eastern	12.7
Asian-American	8.2
Black/African-American	6.8
Native-American	3.2
Mixed Ethnicity	3.2
Place of Birth	
U.S.	46.2
Outside of the U.S.	53.8
Unknown	3.6
Education level	
Less than high school	31.4
High school graduate	20.5
Completed Trade school	5.9
Less than 2 years college	17.7
Bachelor's Degree	13.2
Some Graduate school	1.8
Master's Degree	6.4
Ph.D., M.D. and/or J.D.	1.8
Unknown	1.4
Employment Status	
Full-time	12.7
Part-time	10.4
Not employed	76.9
Unknown	3.6
Marital Status	
Single	6.8
Divorced	12.7
Married	40.5
Widowed	35.9
Divorced and living	
with significant other	0.9
Unknown	3.2

**Table 2:** Demographics Characteristics of Study 2 Sample (*N *= 241)

*Demographic variables*	*Median* *%*
Age	68
Ethnicity	
European-American	28.4
Latina/Hispanic	17.3
Middle Eastern	12.1
Asian-American	9.5
Black/African-American	7
Native-American	< 1.0
Mixed Ethnicity	1.2
Place of Birth	
U.S.	44.6
Outside of the U.S.	48.1
Unknown	1.6
Education level	
Less than high school	19.8
High school graduate	20.9
Completed Trade school	7.4
Less than 2 years college	26
Bachelor's Degree	15.7
Some Graduate school	3.3
Master's Degree	2.9
Ph.D., M.D. and/or J.D.	2.5
Unknown	1.2
Employment Status	
Full-time	17.3
Part-time	10.7
Not employed	72
Unknown	< 1.0
Marital Status	
Single	6.1
Divorced	11.5
Married	50.8
Widowed	30.3
Divorced and living with significant other	1.2
Unknown	< 1.0

### Recruitment Strategies

We recruited participants for both samples using purposive sampling through RAs' connections within their ethnic communities. Recruitment sites included senior centers, grocery stores, and libraries in Los Angeles County. A significant effort was made to identify and minimize barriers to recruitment, with a focus on relevance of questions posed, respect of cultural perspective, and alignment of research goals with those of ethnic minority populations [57,58]. Additionally, we employed face-to-face recruitment to encourage research participation (as most ethnic minority older adults do not respond to advertisements of research studies in the media [59]) and refrained from asking participants to travel to a research center. Recruitment (for both studies) was challenging, as we attempted to recruit as many older women as possible from a vast variety of ethnic backgrounds; several of the women originally contacted were wary of research efforts and uninterested in participating in these two investigations. We allowed for flexible interview scheduling and assigned ethnicity-matched interviewers to establish initial contact and conduct interviews. Moreover, we tailored our recruitment strategies for inclusion of women from all income levels, as socio-economic factors often affect willingness of ethnic minority older adults to engage in research [60]. The utilization of this selection of recruitment tactics and sites maximized the number of ethnic minority participants in the two studies.

### Procedure

Study 1 was conducted in three years (from 2002 to 2005), with two years of data collection, while Study 2 spanned four years (2006-2010), three of which were dedicated to data collection. The last year of funding of each study was dedicated to data entry, verification, and analyses. This federally-funded research was approved by the Institutional Review Board of California State University Northridge. All research assistants (RAs)/interviewers were extensively trained by the first author prior to participating in data collection. Weekly mentoring group meetings were convened to ensure that all of them were well-trained. We asked RAs to start each assessment session by explaining the purpose of the research and addressing any of the respondents' questions. Informed consent was obtained prior to administering the assessment battery. Participants were clearly instructed regarding their right to withdraw from the research at any time, as specified in the consent form. The first part of the assessment procedure involved the solicitation of basic demographic information from each participant and the administration of the brief screener to determine research eligibility. RAs read all items in the test battery aloud and were instructed to complete the assessment in two sessions, with a short break between sessions to minimize fatigue. We also asked them to write all responses to the measures in legible hand-writing in the assessment packets. RAs allowed flexible scheduling of assessment sessions and accommodated location preferences to maximize participants' comfort level. Typically, respondents chose to be assessed in their homes or at community locations including libraries and senior centers. We took great care to exercise patience and preserve the dignity of the older women participating in this research.

### Assessment Tools

RAs were instructed to first administer an original *Screening tool *to verify research eligibility. This short measure employs a combination of items from a standardized tool as well as demographic items (i.e., age and living arrangement) required to determine eligibility. Specifically, it contains a brief portion of the *Survey Psychiatric Assessment Schedule (SPAS) *[61], a 51-item instrument with strong psychometric properties that was used exclusively for screening purposes (as its findings were not included in the data analyses). Our screener comprised only the first five items of the lengthy SPAS, thus excluding the SPAS section "Other survey questions about general health of subject." This choice allowed for the assessment of factors that are potentially indicative of gross cognitive impairment/dementia such as participants' ability to: spell their own name, remember their own birthday, and exhibit an awareness of spatial orientation. RAs were instructed to recruit only women with perfect scores in these three areas.

We used a brief *Demographics list *(created by the first author) to assess variables such as ethnicity, place of birth, education, and income, as well as marital and employment status. An indicator of social support, i.e., the number of people in one's social network, was quantified via the *Single Item Measure of Social Support (SIMSS)*. Although extremely short, this instrument is a strong predictor of morbidity and has good psychometric properties [62]. This single item is comprised of the question "How many people do you have near you that you can readily count on for help in times of difficulty such as watch over children or pets, give rides to hospital or store, or help when you are sick?" Response options are 0, 1, 2-5, 6-10, or more. Responses of 0 or 1 indicate low tangible assistance; 6-10 or more indicate high tangible assistance. Use of this short measure allowed us to avoid utilizing one of the much longer questionnaires on social support, thus rendering the assessment process less cumbersome for older women.

To assess physical and mental QofL, we utilized the *Medical Outcome Study 36-item Short Form Health Survey (MOS SF-36)*. This tool was developed to assess general health status and functional loss [63] and quantifies eight health constructs, namely: 1) limitations in physical activities due to health problems; 2) limitations in social activities resulting from physical or emotional problems; 3) limitations in usual role activities due to physical health problems; 4) bodily pain; 5) general mental health (psychological distress and well-being); 6) limitations in usual role activities because of emotional problems; 7) vitality (energy and fatigue); and 8) general health perceptions. Subtotals for physical and mental health provided a quantification of physical QofL and mental QofL; clinical tests of the validity of this tool have achieved excellent results [64]. We utilized the MOS software to conduct all the analyses relative to physical and mental QofL.

The *Older Women's Social Quality of Life Inventory (OWSQLI) *was used for the first time in the present research. This original measure was developed by the first author; unlike most of the currently available measures on this topic, it is not confounded with health status. As elaborated in the Results, the OWSQLI was created from an original pool of 50 items following an extensive literature review (briefly summarized in the Introduction). This tool was designed to target many culturally-relevant social QofL issues (e.g., feeling cared for by others, feeling valued and respected by others, and contributing to the lives of others). "Others" include family, friends, and the community at large. Item responses are coded on a 7-point Likert-type scale, ranging from "strongly disagree" to "strongly agree." Two sample items are: "I have the power & respect I deserve in my community as an older person with experience & knowledge" and "My children fully understand my social needs."

### Analytic Strategy

We implemented all data analyses through the Statistical Package for the Social Sciences, version 17.0 (SPSS Inc., Chicago, IL). For both studies, we first calculated the sample's descriptive statistics. In Study 1, we conducted an exploratory factor analysis (EFA) of the new tool; moreover, we computed the internal consistency/Cronbach's *α *value for the OWSQLI through a reliability analysis. Additionally, we validated the OWSQLI by relating its scores to those of instruments that measure social support (SIMSS), as well as physical and mental QofL (MOS SF-36). In cases in which a question was not applicable to a given respondent (e.g., for items referring to participant's children, if they were childless, or for questions assuming the availability of at least one friend, if respondents reported that they had none), this question was excluded from the analysis and from any scale construction for that given participant. This is preferable to a listwise deletion that would exclude the person in question from all analyses due to non-applicable responses to a few items. Once the results of the EFA in Study 1 were examined, we conducted a confirmatory factor analysis (CFA) using Study 2 sample. The CFA was implemented specifically to ascertain whether the 22 items that emerged in Study 1 continued to represent a single strong factor from the original 50-item pool in Study 2. Furthermore, we repeated the reliability and validity procedures employed in the previous study and examined (preliminarily) potential ethnic group differences in OWSQLI scores.

## Results

Concerning the EFA in Study 1, our statistical consultant set the criterion of item retention at a factor loading of .32 or higher, which is generally accepted as the minimum value for an item loading on a given factor [65]. A .32 loading is approximately equal to 10% overlapping variance with the other items on that factor. This provided a means to shorten the measure and, consequently, avoid burdening older women with a lengthy instrument in future research in this area. Based on this criterion, 22 of the original 50 items were retained. With 22 items, the mean of the OWSQLI was 39.13 and the standard deviation 20.78. Scores on one item were reversed, as this item is negatively worded (other negatively phrased items did not satisfy the statistical criterion for inclusion). The factor analysis of the OWSQLI obtained robust findings. Based on the 50 original items, the percentage of total score variance explained by one single factor was 44.04% (*λ *= 9.17). Thus, the comparative weight of this factor is strong. Examinations of the Eigen values also pointed to a single-factor solution with a clear drop in size between the first and second factor (9.69 to 1.76). Furthermore, the 7.99% total variance gained in moving to a 2-factor solution was not deemed sufficient, in view of the practical disadvantages of a longer scale and the related greater difficulty in interpretability. Table 3 contains the matrix with the factor loading, which illustrates the degree to which each of the 22 items loaded on one factor, with the most highly loaded items displayed first. To further confirm this 22-item, single factor solution, we conducted a CFA using Study 2 sample. Given the nature of this analysis, Principal Axis Factoring was chosen as the extraction method. All 22 items met the .32 loading threshold with this second independent sample. The OWSQLI yielded a mean of 40.41 and a standard deviation of 19.15. The single factor of the OWSQLI had a strong comparative weight, as it explained a high percentage of total score variance (41.90%, *λ *= 9.22). The matrix with Study 2 factor loading is displayed in Table 4 with the most highly loaded OWSQLI items appearing first.

**Table 3:** Study 1 Factor Loading

*Item* *#*	*Item Content*	*Factor 1*
09	My children fully understand my social needs.	.805
21	My children & grandchildren care about me & how I feel.	.794
08	My children fully understand my physical needs.	.768
30	I have the power & respect I deserve in my family.	.764
50	I feel very loved by my whole family.	.748
13	My family respects me for who I am regardless of my age.	.748
17	My opinions are highly respected in my family.	.747
18	My morals, values & priorities are highly respected in my family.	.729
47	My children have given me the same respect & care I gave my parents.	.713
10	My children fully understand my emotional needs.	.710
40	I am spending a lot of time enjoying myself with my grandchildren.	.705
11	My children fully understand my financial needs.	.661
22	My family looks to me in time of need for advice.	.653
33	I am satisfied with my life in my old age.	.584
42	I feel that I have a lot to contribute to my family.	.584
34	Family traditions are highly respected in my family.	.555
23	I do not feel valued by my family as a source of wisdom.	.466
31	I have the power & respect I deserve in my community as an older person with experience & knowledge.	.463
14	People in the community respect me for who I am regardless of my age.	.448
43	I feel that I have a lot to contribute to my community.	.447
02	I am socially active with friends & in my community.	.379
04	I have gained more respect from others over the years.	.379

Extraction Method: Principal Axis Factoring. One factor extracted. No rotation.

**Table 4:** Study 2 Factor Loading

*Item #*	*Item Content*	*Factor 1*
30	I have the power & respect I deserve in my family.	.873
13	My family respects me for who I am regardless of my age.	.740
17	My opinions are highly respected in my family.	.738
18	My morals, values & priorities are highly respected in my family.	.734
47	My children have given me the same respect & care I gave my parents.	.734
9	My children fully understand my social needs.	.717
31	I have the power & respect I deserve in my community as an older person with experience & knowledge	.696
34	Family traditions are highly respected in my family.	.687
50	I feel very loved by my whole family.	.682
8	My children fully understand my physical needs.	.640
14	People in the community respect me for who I am regardless of my age.	.622
21	My children & grandchildren care about me & how I feel.	.612
10	My children fully understand my emotional needs.	.612
4	I have gained more respect from others over the years.	.586
22	My family looks to me in time of need for advice.	.563
11	My children fully understand my financial needs.	.557
33	I am satisfied with my life in my old age.	.541
23	I do not feel valued by my family as a source of wisdom.	.490
42	I feel that I have a lot to contribute to my family.	.479
40	I am spending a lot of time enjoying myself with my grandchildren.	.453
43	I feel that I have a lot to contribute to my community.	.430
2	I am socially active with friends & in my community.	.363

Extraction Method: Principal Axis Factoring. One factor extracted. No rotation.

Tables 5 and 6 illustrate the item-total statistics for Study 1 and Study 2; the 22 items correlated very well with each other for both samples, which was to be expected, given that they assess facets of one construct, social QofL. In these two tables, we displayed the content of each item next to its original number (out of 50 items).

**Table 5:** Study 1 Item-Total Statistics

*Item #*	*Alpha if* *deleted*	*Wording of item*
23 =	.925	I do not feel valued by my family as a source of wisdom.

02 =	.925	I am socially active with friends & in my community.

04 =	.923	I have gained more respect from others over the years.

43 =	.923	I feel that I have a lot to contribute to my community.

34 =	.922	Family traditions are highly respected in my family.

14 =	.922	People in the community respect me for who I am regardless of my age.

31 =	.921	I have the power & respect I deserve in my community as an older person with experience & knowledge.

42 =	.921	I feel that I have a lot to contribute to my family.

33 =	.921	I am satisfied with my life in my old age.

22 =	.920	My family looks to me in time of need for advice.

40 =	.920	I am spending a lot of time enjoying myself with my grandchildren & children.

50 =	.919	I feel very loved by my whole family.

11 =	.919	My children fully understand my financial needs.

21 =	.919	My children & grandchildren care about me & how I feel.

13 =	.918	My family respects me for who I am regardless of my age.

10 =	.918	My children fully understand my emotional needs.

47 =	.918	My children have given me the same respect & care I gave to my own parents.

18 =	.918	My morals, values & priories are highly respected in my family.

08 =	.918	My children fully understand my physical needs.

17 =	.917	My opinions are highly respected in my family.

30 =	.917	I have the power & respect I deserve in my family.

09 =	.917	My children fully understand my social needs.

**Table 6:** Study 2 Item-Total Statistics

*Item #*	*Alpha if* *deleted*	*Wording of item*
02 =	.927	I am socially active with friends & in my community.

23 =	.924	I do not feel valued by my family as a source of wisdom.

40 =	.924	I am spending a lot of time enjoying myself with my grandchildren & children.

43 =	.924	I feel that I have a lot to contribute to my community.

42 =	.923	I feel that I have a lot to contribute to my family.

11 =	.922	My children fully understand my financial needs.

22 =	.922	My family looks to me in time of need for advice.

33 =	.922	I am satisfied with my life in my old age.

04 =	.921	I have gained more respect from others over the years.

21 =	.921	My children & grandchildren care about me & how I feel.

08 =	.920	My children fully understand my physical needs.

10 =	.920	My children fully understand my emotional needs.

14 =	.920	People in the community respect me for who I am regardless of my age.

34 =	.920	Family traditions are highly respected in my family.

50 =	.920	I feel very loved by my whole family.

09 =	.919	My children fully understand my social needs.

13 =	.919	My family respects me for who I am regardless of my age.

18 =	.919	My morals, values & priories are highly respected in my family.

31 =	.919	I have the power & respect I deserve in my community as an older person with experience & knowledge.

17 =	.918	My opinions are highly respected in my family.

47 =	.918	My children have given me the same respect & care I gave to my own parents.

30 =	.916	I have the power & respect I deserve in my family.

The new measure demonstrated the same high internal consistency in both studies (α = .92, *p *< .001). Moreover, as reported in Table 7 (which contains validity information pertinent to both studies), we conducted a preliminary validity test of the OWSQLI by relating its scores to those on size of social support network, as well as to MOS SF-36 total scores. Good (yet modest, as hypothesized) criterion validity was observed when OWSQLI scores were related to scores on the number of people in the respondents' social network (SIMMS) for both samples. Concurrent validity of the OWSQLI was assessed by comparing its scores in both Study 1 and 2 to those on physical and mental QofL. As expected, correlations among these scales were significant, although modest.

**Table 7:** Criterion and Concurrent Validity for Both Studies

		*OWSQLI* Study 1/Study 2	*SIMSS*	*MOS SF-36* *Physical and Mental Total Scores*
*OWSQLI*	Pearson Correlation	1	-	-

*SIMSS*	Pearson Correlation	.33*/.27**	1	-

*MOS Physical*	Pearson Correlation	.21*/.14*	-	1

*MOS Mental*	Pearson Correlation	.26*/.18*	-	1

* Correlation is significant at the 0.05 level (2-tailed) ** Correlation is significant at the .001 level (2-tailed)

Finally, the samples from Study 1 and Study 2 were combined into one dataset to conduct a preliminary examination of potential ethnic group differences in OWSQLI scores. Combining the datasets was necessary in order to achieve the necessary sample size for mean comparison tests to be meaningful. Even with the significantly larger total sample, it should be noted that only pan-ethnic groupings (African-American, Asian-American, European-American, and Latino-American/Hispanic) rather than specific national-ethnic groupings (e.g. Mexican or Chinese) were possible. We conducted a one-way ANOVA, which was followed by Tukey post-hoc comparisons. Neither the overall omnibus test, *F *(5, 385) = 1.53, *p *= .135, nor any paired comparison between pan-ethnic groups was statistically significant, indicating the general absence of significant pan-ethnic between-group variation in OWSQLI scores.

## Discussion

The findings of Study 1 analyses indicate that the new tool presented herein is characterized by a strong single factor and high internal consistency. These findings were confirmed in Study 2 analyses, whose results were nearly identical to those of Study 1. Furthermore, as expected, the OWSQLI demonstrated adequate criterion validity with a social support indicator, as well as moderate concurrent validity with both physical and mental QofL. These validity results are noteworthy, because they confirm that our measure advances beyond assessment of social support as well as physical and mental QofL to quantify social QofL.

Upon examining specific items loading on the single factor explaining a large part of the OWSQLI's score variance, we discovered that our samples' social QofL scores were affected primarily by satisfying relations with family members. Friends and the community at large were also valued, but their contribution to personal sense of well-being was less critical than familial relationships. This finding substantiates evidence reported in prior literature that the family network is typically the primary social resource available for emotional and financial support in older age, although support from friends and community can provide similar protective effects [66,67]. It also corroborates the notion, reinforced in a recent literature review [68], that family ties are particularly important for non-European-American older adults (as most of our participants had an ethnic minority status). Interestingly, the notion of respect appears in the items that have some of the highest loadings in the scale, which highlights the importance of respect and of one's role in the family in older age, especially considering compelling research relating family role to mortality in older age [69-71].

The fact that issues such as being loved and cared for by family and friends were strongly related to total OWSQLI scores suggests that it is imperative for mental health providers to encourage older patients to focus on ties with family and friends in order to strengthen such ties and, in turn, possibly enhance biopsychosocial functioning in later life. In particular, the "friends-related" finding offers supportive empirical evidence for Yeh and Lo's recommendations to provide incentives within communities to engage older adults in social activities and enhance their social contacts and resources [72]. We also discovered that a sense of belonging and maintenance of status as a contributing family member is positively linked to older women's social QofL. These findings complement prior empirical evidence demonstrating that supportive relationships and activities that are perceived as important help maintain older women's self-esteem and reduce stress [73], and that social contact and frequent social interaction produce higher life satisfaction in later life [74].

Another consistent result across both studies was that perceived size of social network was significantly related to OWSQLI scores, again indicating that the amount of available help (i.e., individuals who are perceived as willing and able to provide practical assistance when in need) is an important factor with regard to older women's social QofL. These findings are corroborated by prior literature on this topic [23]. Our results concerning the significant relationship between physical health and social QofL are also consistent with prior empirical evidence on this topic linking social resources to better physical health and a lessened impact of chronic disease [21]. Moreover, the significance of the relationship between mental and social QofL corroborates the findings of other researchers [27,30]. As a whole, the results of our two studies indicate that the new measure of social QofL is a psychometrically sound instrument with desirable properties for utilization with older women from diverse ethnic backgrounds. Moreover, the items comprising the OWSQLI employ rather simple language, which is a strength of the measure, given the modest educational level of both samples.

These two investigations have several limitations that could be addressed in future studies. Among them, our findings do not imply causation, because their design was correlational. Longitudinal research is needed to verify the present results. Also, the OWSQLI was not validated against a previously established socially-related QofL instrument. Yet, because, to our knowledge, this is the first tool of its kind, such a limitation was unavoidable. However, it would have been ideal to validate the OWSQLI against a measure of social support that was more lengthy and in-depth than the SIMSS. A focus on keeping the assessment battery short (to reduce fatigue among research participants) prevented us from using a lengthier social support instrument. In future studies, researchers could compare scores on the OWSQLI to those on other well-established social support measures such as the Q-LES-Q [49].

Another limitation of our research was that we recruited women exclusively; men's social QofL should also be studied using our tool, as the latter could be validated and modified accordingly for use with ethnically diverse older men. Furthermore, a few of the items in the OWSQLI are worded somewhat vaguely, especially given that the scale was administered to women as young as 60 (many of whom were still working). For instance, the meaning of "... regardless of my age" could be unclear; we meant that, no matter how old the participant was, she felt respected (or not) by the people in her community and by her family members. Having a few items with words that could be assigned different meanings is a shortcoming of most of the aforementioned tools. Special attention should be paid to clarifying the meaning of unclear items and encouraging research participants to raise questions if items are not understandable (although, in our research, none of the respondents asked to clarify the meaning of any of the scale's items). Moreover, the sample sizes of specific ethnic groups did not allow for the testing of national groups -- although we did attempt to unveil different social relationship patterns via mean comparisons across pan-ethnic groupings, but found no significant differences in mean OWSQLI scores. Interested researchers could identify how older adults from different ethnic minorities respond to this tool and develop culture-specific norms based on their findings. In this regard, the meaning of words such as "having power in one's family" could vary across ethnic groups. Ideally, these issues should be explored via in-depth qualitative interviews (in addition to administering paper-and-pencil measures) to clarify item comprehension and assignment of meaning to complex concepts like power. Including collection of qualitative data to study social QofL would allow a more in-depth study of this topic via mixed methods procedures.

Concerning additional limitations of these two research endeavors, all participants in both studies were fluent in English; interested investigators should consider using and validating this new tool in other languages. Also, our two cohorts were not based on random community sampling, so our findings may not be fully generalizable to our catchment area. However, about half of both our samples reported holding a high school degree at best, similarly to findings on the general Los Angeles County population (47.3%) [75]. Judging from those statistics, the two samples utilized in this research are possibly representative of their catchment area. Nonetheless, our results could be due, at least partially, to variables not assessed herein such as medication use and/or acculturation. Further psychometric evaluation, such as testing for differences on a subset of the items of the OWSQLI (e.g., family items) across a stratified sample, may be warranted, but was beyond the scope of this preliminary test of our measure. Additionally, the exclusive use of self-rated measures, although implemented in most of the aforementioned investigations, is a limitation. Overall, in spite of multiple limitations, our studies represent a necessary step in the direction of properly quantifying the social QofL of ethnically diverse older women, thus making a needed contribution to the growing area of ethnogeriatric psychometrics.

## Conclusions

In our research, we intended to start addressing a gap in the available geriatric literature concerning the need to develop an instrument to assess social QofL in older age independent of physical QofL. Our research findings with two non-clinical samples of community-dwelling older women from diverse ethnic backgrounds reinforce the notion that QofL in older age is truly multi-dimensional and must be carefully quantified through domain-specific tools that are sensitive to older adults' complex needs and resources. The evaluation of physical and mental QofL alone is insufficient for a comprehensive quantification of well-being in later life. As testing of our tool achieved promising results, clinicians who intend to assess social QofL in older women should consider adding this new measure to their assessment battery. This would allow them to gain a deeper understanding of social areas in which their patients are lacking support, as well as social areas of particular strength. Both could be enhanced via interventions such as individual therapy to increase psychosocial resilience, family therapy, and implementation of programs targeting social activities that the older patients in question find personally meaningful. Interested investigators should test the psychometric properties of the OWSQLI further; currently, in our laboratory, we are collecting data utilizing this measure on ethnically diverse older men in order to refine this tool for use with older male populations.

As a final consideration, researchers interested in clinical applications of the OWSQLI could relate its scores to those on adjustment to challenges commonly experienced in older age, including chronic impairments such as vision problems. Utilization of the OWSQLI, given its "family and friends" focus, could be ideal with clinical populations similar to the one targeted in a recent study [76] in which family was found to provide social support during the initial adaptation of older individuals to vision impairment, while friends provided support in long-term adaptation. Given the differences between our studies' non-clinical population and the clinical one in question, the factor structure of the OWSQLI for patients with progressive physical impairments could look significantly different (at different stages of their adaptation to the visual impairment) than the structure obtained herein.

## Competing interests

The authors declare that they have no competing interests.

## Authors' contributions

LL, the principal investigator, created the OWSQLI, designed the two studies, conducted Study 1 analyses, and wrote most of the paper. MLB wrote some parts of the literature review and a first draft of a portion of this paper. IB wrote some parts of the literature review, contributed to the writing of all sections, verified the accuracy of the methodological parts of the paper, and conducted Study 2 data analyses. All authors read and approved the final manuscript.

## Pre-publication history

The pre-publication history for this paper can be accessed here:

http://www.biomedcentral.com/1471-2318/11/60/prepub
